# Bacteria Isolations from Broiler and Layer Chicks in Zambia

**DOI:** 10.1155/2012/520564

**Published:** 2012-09-03

**Authors:** Hetron Mweemba Munang'andu, Swithine Hameenda Kabilika, Oliver Chibomba, Musso Munyeme, Geoffrey Munkombwe Muuka

**Affiliations:** ^1^Section of Aquatic Medicine and Nutrition, Department of Basic Sciences and Aquatic Medicine, Norwegian School of Veterinary Sciences, Ullevålsveien 72, P. O. Box 8146 Dep, 0033 Oslo, Norway; ^2^Central Veterinary Research Institute, Ministry of Agriculture, Food, and Fisheries, P.O. Box 33790, Balmoral, Lusaka 10102, Zambia; ^3^Department of Disease Control, School of Veterinary Medicine, University of Zambia, P.O. Box 32379, Lusaka 10101, Zambia

## Abstract

Chick mortality (CM) is one of the major constraints to the expansion of the poultry industry in Zambia. Of the 2,829 avian disease cases submitted to the national diagnostic laboratory based at the Central Veterinary Research Institute in Lusaka between 1995 and 2007, 34.39% (973/2,829) were from CM cases. The disease accounted for 40.2% (218,787/544,903) mortality in the affected flocks with 89.6% (196,112/218,787) of the affected birds dying within seven days. Major bacteria species involved were *Escherichia coli*, *Salmonella gallinarum*, and *Proteus species* being isolated from 84.58%, 46.15%, and 26.93% of the reported CM cases (*n* = 973), respectively. Detection of *Salmonella typhimurium*, *Salmonella enteritidis*, and *Salmonella dublin* indicates that poultry has the potential of transmitting zoonotic pathogenic bacteria to humans. The proportion of *Salmonella gallinarum* reactors in the adult breeding stock was generally low (<0.5%) throughout the study period although its prevalence in CM cases was correlated (*r* = 0.68, *P* < 0.011) with seroprevalence of the same pathogen in the adult breeding stock. Given that the disease accounts for a large proportion of the avian diseases in Zambia as shown in the present study (34.39%, *n* = 2,829), it is imperative that an effective disease control strategy aimed at reducing its occurrence should be developed.

## 1. Introduction

Poultry production has steadily expanded in Zambia from an estimate of 16 million birds in 2000 to about 26 million by 2008. By 2008, it is estimated that 1.3 million chickens and six millions eggs were produced every month [1]. It is likely that the production has increased further in the last four years. The expanding poultry production is not only increasing the protein source for the local demand but is also increasing the export of poultry products to neighboring countries. In Zambia the largest proportion of poultry farmers are small scale commercial growers although the supply of day old chicks is limited to a few hatcheries. However, the major constraint to the expansion of the poultry industry in Zambia is the presence of diseases which include chick mortality (CM) which is characterized by omphalitis and yolk sac retention occurring during the first few days after hatching. The devastating impact of this disease has been reviewed by different authors [2–4] in different countries although its impact and factors responsible for its persistence in Zambia have not been elucidated.

Previous studies carried out in Zambia focused on isolation and identification of bacteria from table eggs [5], dead-in-shell embryos [6], and adult birds at point of sale [7]. Environmental sampling of processing plants for adult birds at point of sale has also been studied [8]. Bacteria isolations from these studies indicate that transmission from adult birds to eggs could be responsible for the transmission of bacteria pathogens within flocks [9]. Surveillance on different poultry diseases in Zambia has been carried out [9, 10] although the impact of CM on the poultry industry and the pathogens associated with the disease has not been investigated. In the present study we reviewed the bacteria species associated with CM in Zambia for the period 1996 to 2008 and factors responsible for the persistence of the disease are herein highlighted. The idea was to identify the pathogens associated with chick mortality in order to obtain baseline information that would help veterinarians develop disease control strategies that would help reduce occurrence of the disease in the poultry industry. The significance of poultry as a source of zoonoses transmissible to humans and the importance of improving the control of avian diseases is herein discussed.

## 2. Methods and Materials

### 2.1. Diagnostic Questionnaire

Chicken samples from poultry farmers who took their birds for diagnosis at the national diagnostic laboratory based at the Central Veterinary Research Institute (CVRI) in Lusaka constituted the study material for the present investigation which covered the period 1996–2008. Diagnostic report forms were filled in upon receiving the samples in order to obtain background information. The information obtained included the name of the farmer, age, breed, and source of the birds. The date the birds were bought from the hatchery, total number of birds in the flock, number affected, clinical observations, and the number of birds that died of the disease was also recorded. Management data obtained included feeding regimes and the farming system which was classified as broiler, layers, or traditional farming system. Disease control regimes used were also recorded mainly focusing on the treatment and vaccination records. Laboratory data included the number of samples examined, date samples were received and a report of the findings.

### 2.2. Laboratory Examination

Postmortem examination was carried out on the chicks submitted for diagnosis. Yolk sac and visceral organs were aseptically collected for bacteriological examination. Inocula from yolk sacs and viscera were made on various selective and differential media for bacteria isolation. All inoculated media were incubated at 37°C for 24–48 hrs. For enrichment, samples collected for salmonella isolation were inoculated in selenite broth and incubated at 37°C for 24 hrs followed by inoculation on MacConkey agar. All media and agar were prepared according to manufacturer's recommendations (DIFCO, UK). After 24–48 hrs of incubation at 37°C, colonies were examined for cultural and morphological properties on growth media by determining the size, shape, elevation, edges, surface, and color of the colonies on agar. Other changes examined were the ability of bacteria colonies to cause hemolysis in the case of samples cultured on blood agar. Thereafter, smears on slides were prepared from the colonies for gram staining to classify the isolates into gram positive or gram negative under a light microscopy at ×100 magnification under oil immersion. Gram staining was carried out according to the method described by Merchat and Packer [11] and Cheesbrough [12]. This allowed for further analysis of the morphological properties and classification of bacteria as cocci, coccobacilli, bacilli, or spriochaetes. Bacteria colonies were subcultured to obtain pure colonies for identification as most plates contained mixed colonies with different properties on first culture. For species identification, pure colonies were subjected to standard biochemical tests that included indole, voges proskauers, methyl red, and citrate utilization tests while carbohydrate fermentation tests included glucose, lactose, arabinose, lactose, sorbitol, sucrose, and manitol [13]. Motility tests aimed at differentiating motile from nonmotile bacteria was performed according to the method described by Cowan [14]. Salmonella isolates were further subjected to polyvalent somatic antigen classification using salmonella polyvalent O and H antisera (Oxford Company Limited Basingstoke, Hampshire, England). Overall, identification of bacteria isolates was based on their growth characteristics, morphology, staining reaction, motility, and biochemical and carbohydrate fermentation tests as described elsewhere [13].

### 2.3. Breeder Stock Serological Tests

Screening of *Salmonella gallinarum* antibodies of the breeder stocks at hatcheries that supply day-old-chicks to layers and broilers farmers was carried out for the study period 1996–2008. A standard *Salmonella gallinarum* strain was propagated in nutrient agar at 37°C, harvested, and processed for use as antigen in the serum agglutination test as described elsewhere [15]. The antigen was characterized by using polyvalent “0” and “H” Salmonella antisera as described by the manufacturer (Oxford Company Limited Basingstoke, Hampshire, England). Serological tests of the breeder/parent stock at hatcheries was carried out by mixing 50 *μ*L of the antigen with an equal volume of the test sera on a clean white tile marked in squares of 3 × 3 cm^2^. After constant rotation for 2 minutes the mixture was examined for the presence of agglutination. The test was carried out on 5–10% of the breeders in the hatcheries. In hatcheries where reactors were detected, detailed investigations which included slaughter of the seropositive birds followed by postmortem examination, bacteria isolation and identification were carried out. As a control measure, hatcheries diagnosed positive of salmonellosis were closed followed by application of zoo-sanitary control measures.

### 2.4. Data Analysis

Data from diagnostic reports and serological screening tests were entered in excel sheets (Microsoft Excel) and were later analyzed using STATA version 10. The Spearman's rank correlation coefficient test was used to measure the correlation between seroprevalence of *Salmonella gallinarum* reactors in the adult breeding stock in the hatcheries and occurrence of the same pathogen in CM cases on poultry farms.

## 3. Results

### 3.1. Data Analysis

**Figure 1:**
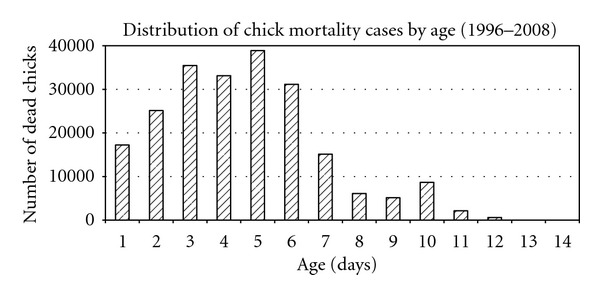
It shows the distribution of chick mortalities (CM) cases by age for the period 1996–2008.

Of the 2,829 poultry disease cases submitted for diagnosis at CVRI during the period 1996–2008, 1098 were diagnosed positive of CM (Table 1). Our findings indicate that CM accounted for a third (34.39%, *n* = 2,829) of the avian disease cases diagnosed at the Central Veterinary Research Institute (CVRI), which is the national laboratory for diagnosis of animal diseases. The disease accounted for 40.2% (*n* = 218,787) mortality in the affected flocks. Of 1,098 CM positive cases, 62% were from broiler-farmers while 30.1% were from layer-farmers and only 1.8% was from traditional farmers. Our findings indicate that 89.6% (*n* = 218,787) of the affected chicks died within the first week (Figure 1). Table 1 reflects the mean size of the affected flocks in each year and the proportion of CM cases in relation to the total number of avian cases submitted for diagnosis. Of the 973 CM cases examined, 16.8% (164/973) were from farmers that had treated their chicks with antibiotics. The main antibiotics used were oxytetracycline 23.5% (*n* = 164), penicillin 18.9% (*n* = 164), furazolizone 13.5% (*n* = 164), and 10.2% (*n* = 164) furaltodone.

### 3.2. Pathological and Bacteriological Findings

**Figure 2:**
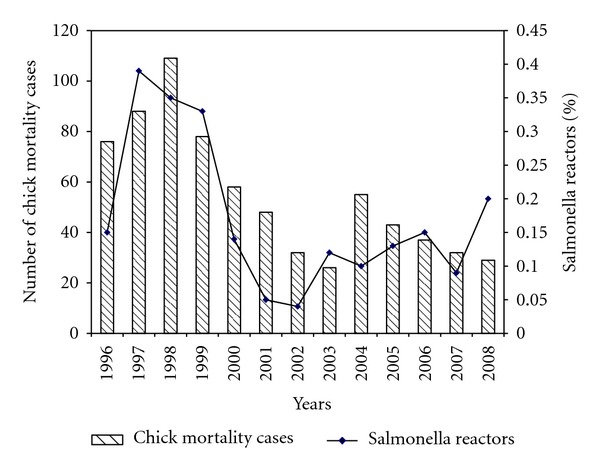
Shows the distribution of *Salmonella gallinarum* reactors in the hatcheries and the number of *Salmonella gallinarum* cases associated with chick mortality (CM) for the period 1996–2008.

Clinical observations indicated that affected chicks appeared drowsy, had distended abdomens, and were clustering around heat sources. Mortalities were high within the first seven days (89.6%, *n* = 218,787) after purchase from hatcheries and chicks that survived showed signs of stunted growth. Pathological observations were mainly characterized by inflamed navels, failure of the navel to close, unabsorbed yolk sacs, and peritonitis. Subcutaneous edema was a common feature with some chicks having bluish discoloration of the abdominal muscles around the navel. Cases were either reported as yolk sac retention or omphalitis and all birds that died with these characteristics were classified as CM for the purpose of this study. Bacteriology results showed mixed infections involving *Escherichia coli*, *Salmonella gallinarum*, and *Proteus species* 13.2% (*n* = 973), *Escherichia coli* and *Salmonella gallinarum* 10.1% (*n* = 973) while single colony infections were only recorded from *Escherichia coli* 25.7% and *Salmonella gallinarum* 4.3%. *Escherichia coli* was the most prevalent bacteria isolated from 84.58% (*n* = 973) of the reported cases followed by *Salmonella gallinarum* 46.15% (*n* = 973) and *Proteus species* 26.93% (*n* = 973) (Table 2). We observed a high correlation (*r* = 0.68, *P* < 0.011) between the occurrence of *Salmonella gallinarum* in CM cases and the number of *Salmonella gallinarum* adult breeding stock reactors in the hatcheries indicating that occurrence of *Salmonella gallinarum* among the layers and broiler farmers could be linked to increase in the number of adult breeding stock reactors on the hatcheries.  Figure 2 shows the percentage of adult breeding stock reactors in the hatcheries and chick mortality cases on layer and broiler farms for the period 1996 and 2008.

## 4. Discussion

Thus far, there have been few studies carried out on avian diseases in Zambia despite the expanding poultry industry. The present study shows that CM is one of the major constraints to poultry production considering it accounted for 34.39% (*n* = 2,829) of the avian diseases reported for diagnosis at the CVRI, which is the national diagnostic laboratory, for the period 1996–2008. As shown in Table 2 the mean annual mortality of CM was estimated at 40.2% (218,787/544,903) in the affected flocks indicating a significant economic loss for the affected farmers. Hence, it is imperative that control measures should be put in place to reduce the occurrence of the disease.

Previous studies carried out in Zambia focused on the prevalence of bacteria isolates from table eggs [16, 17], dead-in-shell embryos [18–20], adult birds at point of sale [7, 21, 22], and environmental samples collected from hatcheries [20]. It is interesting to note that most bacteria species isolated from these studies were also isolated in the present study suggesting that these pathogens are transmitted from adult birds in the hatcheries to the eggs subsequently leading to CM with *Escherichia coli*, *Proteus* spp., and *Salmonella* spp. being the most prevalence bacteria species. Although we observed a low seroprevalence of *Salmonella gallinarum* in the hatcheries (<0.5%) the number of reactors detected were correlated with the occurrence of *Salmonella gallinarum* in CM cases among the poultry farmers. Hang'ombe et al. [5] reported that the prevalence of *Salmonella* species was higher in the yolk sac (22.82%, *n* = 2400) than the shell membranes (4.76%, *n* = 2400) suggesting that the the yolk sac was a more favorable site for bacteria propagation than the shell membranes. It is likely that the environmental contamination of the hatcheries reported by Tuchili et al. [23] leads to infection of the eggs with most bacteria localizing in the yolk sac during the incubation process. Eggs that fail to hatch end up as dead-in-shell embryos retaining the infecting bacteria in the yolk sac while others hatch with failure of the navel to close as a result of bacterial infection leading to yolk sac retention and omphalitis. Hence, pathogenic bacteria species detected in environmental samples obtained from hatcheries and processing plants are similar to those detected in the dead-in-shell embryos and CM cases observed in the present. This would account for reasons why Sato et al. [9] were able to trace outbreaks of salmonellosis to the hatcheries.

Isolation of *Salmonella typhimurium* and* Salmonella enteritidis* (Table 2) poses a significant public health threat because it is indicative that hatcheries and poultry farms in Zambia have zoonotic organisms have the potential of entering the food-chain. Hang'ombe et al. [22] isolated *Salmonella enteritidis* from pooled table eggs and chickens carcasses processed for human consumption indicating that eggs and chickens are potential sources of infection to humans. Dube [24] reported a high carrier rate of *Salmonella* spp. and* Shigella* spp. among food handlers while *Escherichia coli* and various *Salmonella* spp. inclusive of *Salmonella enteritidis* and *Salmonella typhi* have been associated from various diseases infecting humans in Zambia [25–28]. Put together, these studies indicate that zoonotic pathogens found in poultry have been associated with various diseases in humans. Hence, it is imperative that disease control strategies should not only focus on reducing the occurrence of bacterial infections in poultry, but should include the need to reduce the threat of zoonotic pathogens from infecting humans.

Although 16.8% of the farmers used antibiotics in treating CM cases, it is likely that the high mortality which occurs within a few days after the chicks are hatched might not allow enough time for antibiotic to clear the infection. There is a need for detailed investigations on the use of antibiotics in CM-affected flocks in order to determine the correct timing needed to save infected birds from dying. The decline in the number of cases observed in the last five years of the study period could be attributed to a proactive approach initiated by the Department of Veterinary Services in sensitizing poultry farmers and hatchery owners on a farmer based participatory approach in controlling poultry diseases. This move was supported by the formation of the Poultry Producers Association (PPA) which calls for seminars and conferences aimed at educating poultry farmers on farming practices that minimize disease transmission. Current control measures do not permit hatchery owners to vaccinate their breeder stock against Salmonellosis because this would obscure the monitoring of breeding stocks using serological tests. Overall, our findings and results obtained from previous studies [5–7, 23] indicate that *Escherichia coli* followed by *Salmonella gallinarum* and *Proteus* spp. are the most prevalent bacteria species isolated from CM, environmental contamination in processing plants, in table eggs, dead in shell embryos, chickens processed for market sales, and hatcheries. Given that CM accounted for 34.39% (*n* = 2,829) of the poultry disease cases for the period 1996–2008 diagnosed at CVRI, it is evident that CM is one of the major constraints to the expansion of the poultry industry. Therefore, we strongly advocate for stringent disease control measures aimed at reducing the occurrence of this disease in Zambia.

## Figures and Tables

**Table 1:** Proportion of CM mortality cases for the period 1995–2008.

Parameter	Year													
	1996	1997	1998	1999	2000	2001	2002	2003	2004	2005	2006	2007	2008	Totals
Total avian disease cases	512	454	644	489	383	211	213	260	152	121	96	87	66	2,829
Total number of CM cases	135	123	166	169	112	56	48	109	78	58	38	32	26	973
Proportion of CM cases (%)	26,37	27,09	25,78	34,56	29,24	26,54	22,54	41,92	51,32	47,93	39,58	36,78	39,39	34,39
Mean flock size (CM farms)	301,95	513,18	521,20	451,13	394,57	350,48	266,94	663,86	572,51	827,84	354,08	435,16	354,38	560,02
Total in affected flocks	40763	63121	86520	76241	44192	19627	12813	72361	44656	48015	13455	13925	9214	544903
Mortality due to CM	19364	38626	45053	18101	16261	14101	4564	13177	14162	19111	4778	8777	2712	218787
CM mortality %	47,5%	61,2%	52,1%	23,7%	36,8%	71,8%	35,6%	18,2%	31,7%	39,8%	35,5%	63,0%	29,4%	40,2%

**Table 2:** Bacterial isolations from chick mortality cases 1995–2008.

Bacteria isolations	Year													Totals	%
	1996	1997	1998	1999	2000	2001	2002	2003	2004	2005	2006	2007	2008
Total CM cases	135	123	167	169	112	56	48	109	78	58	38	32	26	973	100
*Escherichia coli*	112	96	101	122	86	44	38	81	51	39	21	16	16	823	84,58
*Salmonella gallinarum*	72	44	67	42	31	15	27	65	22	15	15	19	15	449	46,15
*Salmonella typhimurium*		1		3			2	2		1		2		11	1,13
*Salmonella enteritidis*			2	6		3	12		6		1			30	3,08
*Salmonella dublin*		2		1				1						4	0,41
*Salmonella* species	12	10	21	2	19	7	2	8	3		1		1	86	8,84
*Proteus* species	23	36	33	46	34	19	15	23	7	10	2	8	6	262	26,93
*Proteus morgani*	1		3	8		3				2	1			18	1,85
*Proteus urabilis*			3					2				3		8	0,82
*Psedomonus aeroginosa*	8	6	2	10	5	6	1	1	6	2	3	1		51	5,24
*Klebsiella* species	1	2	1	3	1	1	1	2	1	2	1	1	1	18	1,85
*Klebsiella ozanenae*	2											2		4	0,41
*Moraxiella* species	1	2	3		2		2	1		4	1			16	1,64
*Streptococcus* species	11	2	4	1		1					2			21	2,16
*Staphylococcus* species	3	2								3	3			11	1,13
*Actinobacillus* species	2													2	0,21
*Actinobacillus anitratus*		2												2	0,21
Anthracoids	7	2												9	0,92
*Shigella* species		1							2		2			5	0,51
*Bordetella parapertusis*	1													1	0,10
Yeast cells	16	13	20	9	6	17	4	6	10	8	1	5	2	117	12,02
*Enterobacter* species		3			2				3				2	10	1,03
*Corybacteria* species	1						1							2	0,21

## Abbreviations

CM: Chick mortality
CVRI: Central Veterinary Research Institute.
